# Advances in electrochemical sensors based on nanomaterials for the detection of lipid hormone

**DOI:** 10.3389/fbioe.2022.993015

**Published:** 2022-09-09

**Authors:** Tingting Zhang, Xin Du, Zhenguo Zhang

**Affiliations:** Shandong Provincial Key Laboratory of Animal Resistance Biology, College of Life Sciences, Shandong Normal University, Jinan, Shandong, China

**Keywords:** lipid hormone, electrochemical biosensor, detection principles, research progress, nanomaterials

## Abstract

Lipid hormone is produced by highly differentiated endocrine cells and directly secretes into the blood circulation or tissue fluid to act as information transmission. It influences the physiological functions of the human body by controlling the metabolic processes of multiple tissue cells. Monitoring the levels of lipid hormone is of great importance for maintaining human health. The electrochemical sensor is considered as an ideal tool to detect lipid hormone owing to its advantages such as quick response, convenience and low economic costs. In recent 3 years, researchers have developed various electrochemical sensors for the detection of lipid hormone to improve their sensitivity or selectivity. The use of nanomaterials (such as carbon nanomaterials, precious metal and polymer) is a key research object and a breakthrough for improving the sensing performance of electrochemical sensors for detection of lipid hormone. This paper reviews and discusses the basic principle, nanomaterials, actuality and future development trend of electrochemical sensors for the detection of lipid hormone in the past 3 years.

## 1 Introduction

A range of hormones, including steroid and fatty acid derivative hormone, are classified as lipid hormone because of their lipid chemical makeup. Sex hormone, adrenocortical hormone and vitamin D (VD) are three primary subtypes of steroid hormones. The three primary types of fatty acid derivative hormones are prostaglandin, leukotriene, and iso-prostaglandin. Although the human body contains relatively little of these compounds, they can have a big physiological impact such as regulating menstruation (Critchley et al., 2020), preventing anxiety (McHenry et al., 2014) and regulating blood sugar levels (Vargas et al., 2020). Hormonal imbalances can be harmful to human health (Adegoke et al., 2021). Levels of all lipid hormones are significant diagnostic indicators for possible disease status (Pelizzaro et al., 2021). Thus, early detection by measuring the level of hormone has important significance for the identification of human diseases and the advancement of medical science.

Currently, the traditional analysis methods of lipid hormone are high-performance liquid chromatography (Ozgocer et al., 2017), liquid chromatography-mass spectrometry (Zhang et al., 2021), chemical luminescence method (Abdulsattar and Greenway, 2019) and enzyme-linked immunosorbent assay (Yin et al., 2019). However, these traditional detection techniques have the limitations of complex preparation steps, high analysis costs and time-consuming. Under these circumstances, electrochemical biosensors offer certain benefits over conventional analytical techniques, including simplicity, portability and quick detection. (Felix and Angnes, 2018) which has been applied in the fields of drug research, clinical diagnosis, food safety testing, environmental monitoring and other fields (Aydin et al., 2019). The types of the electrochemical sensor of lipid hormone that have been reported including electrochemical immunosensors (Mathew et al., 2020), electrochemical aptamer sensing (Tang et al., 2022) and electrochemical molecular imprinted polymer (MIP) sensors (Rebelo et al., 2020). Many nanomaterials including graphene, carbon nanotubes, gold nanoparticles (AuNPs) and adapters were applied in the preparation of the sensor.

This paper mainly reviews the application of electrochemical biosensors in lipid hormone detection in the past 3 years, including various kinds of detection principles, the application of nanomaterials, performance and advantages of these electrochemical sensors. We also summarize the future direction of electrochemical biosensors in lipid hormone detection, containing the challenges of current techniques and emerging applications.

## 2 Principle of electrochemical sensors for detection of lipid hormone

The biosensor is an instrument that uses immobilized bio-sensitive materials (antibodies, enzymes, receptors, cells, microorganisms, nucleic acids, etc.) as biometric elements to recognize the required target molecule and converts the analyte concentration into electrical signals for detection (Ronkainen et al., 2010). In general, an electrochemical biosensor has three components including biometric elements, sensors and electronic systems.

Nanomaterials are often applied to modify the electrode to amplify the detection signal. Commonly used nanomaterials include carbon nanomaterials (such as carbon nanotubes, carbon quantum dots and graphene), precious metal nanomaterials [such as gold (Au) and silver (Ag)], metal oxides (such as copper oxide and titanium oxide), polymer nanomaterials (such as MIP and conducting polymers) and biological nanomaterials (such as adapters) (Raza et al., 2021). Furthermore, the synergistic effect of multi-component nanomaterials can provide more obvious additional advantages.

**FIGURE 1:**
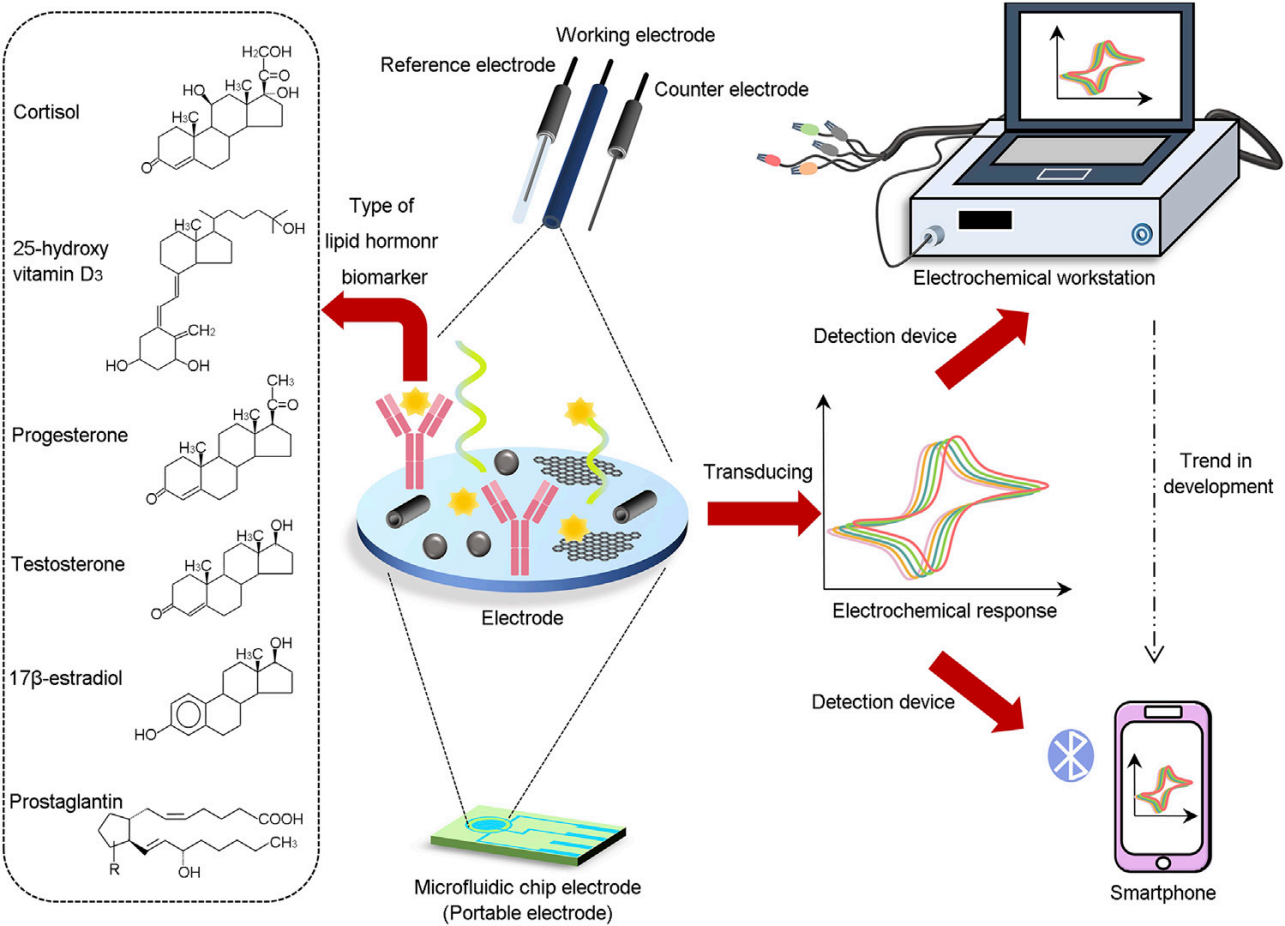
Schematic representation of the electrochemical biosensor for detection of lipid hormone.

The common detection methods of electrochemical sensors are cyclic voltammetry (CV), differential pulse voltammetry (DPV), square wave voltammetry (SWV) and electrochemical impedance spectrum (EIS). These detection methods all have their own uses and characteristics: CV may be used to verify both the reversibility of a reaction and the existence of intermediates in redox processes. With DPV and SWV, target analytes can even be detected at picomolar or femtomolar concentrations (Goud et al., 2021). EIS may be used to determine if the electrode/electrolyte interface impedance changes when the target is bound to the surface-immobilized biorecognition element. It has broad measured range and high detection stability as its main traits (Li et al., 2019). With the progress of electrochemical sensors, the detection method has gradually developed from desktop to portable and wearable biosensors (Samson and Koh, 2020). The schematic representation of the electrochemical biosensor for detection of lipid hormone is shown in Figure 1.

## 3 Research on the electrochemical sensor of lipid hormone

Electrochemical sensors for the detection of lipid hormone have had significant success recently. A summary of electrochemical sensors for the detection of lipid hormone is listed in Table 1.

**TABLE 1:** A summary of electrochemical sensors for the detection of lipid hormone.

Lipid hormone	Nanomaterials	Electrochemical Method	Sample	Linear range (nM)	Detection limit (nM)	Reference
progesterone	poly-L-serine/AuNPs/MWCNTs	CV, EIS	human serum	1–2000	200	Naderi and Jalali, (2020)
progesterone	MGO	DPV, CV	tap water	1 × 10^−4^–1000	1.5 × 10^−4^	DishaKumari et al. (2021)
progesterone	Fe_3_O_4_@SiO_2_@TiO_2_–NH_2_–aptamer–cDNA	EIS	milk	0.001–6	3 × 10^−4^	Li et al. (2020)
progesterone	BSA/aptamer/GQDs-NiO-AuNFs/f-MWCNTs	DPV	human serum, pharmaceutical products	10–100	1.8 × 10^3^	Samie and Arvand, (2020)
progesterone	aptamer-Au-CuO-Cu_2_O/progesterone/Ab/CDs-GO	photoelectrochemical	human serum	0.5–180	0.17	Zhu et al. (2020)
17β-estradiol	NiFe_2_O_4_-MC	CV, DPV, SWV	drug tablets	20–566	6.88	Tanrikut et al. (2020)
17β-estradiol	CDs-PANI	CV	human serum, water	1-1 × 10^5^	43	Supchocksoonthorn et al. (2021)
17β-estradiol	poly(β-CD)/AF1-ADA/ON1/AF2-Au	DPV, EIS	milk	0.001–10	7 × 10^−7^	Chang et al. (2021)
17β-estradiol	EIPs/WS_2_	CV	eel serum	0.37–3,671.34	2.08 × 10^−7^	Lee et al. (2020)
17β-estradiol	MIP/CB	DPV	river water	100–2.3 × 10^4^	30	da Silva and Pereira, (2022)
17β-estradiol	MIP	CV, SWV	real water	0.01–100	0.06	Regasa and Nyokong, (2022)
testosterone	TIECP	CV	human urine	0.35–346.72	∼pM	Liu et al. (2020)
testosterone	Ab/pBDBT	amperometric	synthetic urine and serum	34.67–1733.58	58.08	Bulut et al. (2020)
cortisol	ALP/1-NPP	CV	human serum	0–1091.46	63.03	Vargas et al. (2020)
cortisol	AuNPs/MWCNTs	CV, DPV	human sweat	2.73 × 10^−6^-273	8.19 × 10^−7^	Liu et al. (2021)
cortisol	DTSP/AuNPs/graphene	CV	artificial sweat	0.03–212.87	0.03	Naik et al. (2021)
cortisol	MIP	CV	artificial sweat	10–1000	0.2	Tang et al. (2021)
cortisol	poly(GMA-co-EGDMA)/CNC/CNT	CV, EIS	human sweat	27.29–180.09	5.46 ± 1.09	Mugo and Alberkant, (2020)
cortisol	MWCNTs/CMK-3/AgNPs	CV, DPV	human saliva	2.7 × 10^−4^–2.7 × 10^−2^	2.5 × 10^−4^	Huang et al. (2021)
cortisol	MIP-aptamer/N-CQDs-FG	CV, DPV, EIS	human saliva	0.001-10	3.3 × 10^−4^	Yu et al. (2022)
VD	graphene/Nafion	CV, SWV, EIS	food	113.64–5681.82	35.79	Thangphatthanarungruang et al. (2020)
25(OH)D_3_	BSA/Ab-25(OH)D_3_/EDC-NHS/GCN-β-CD@AuNPs	CV, DPV, EIS	serum	0.25–1247.97	0.03	Anusha et al. (2022)
25(OH)D_3_	Ab/Cys/Au/MoS_2_	CV, DPV, EIS	human serum	0.0025–249.59	9 × 10^−4^	Kaur et al. (2021)
VD_3_	GQD-Au/aptamer-VD_3_	EIS	human serum	1–500	0.7	Wadhwa et al. (2020)
25(OH)D_3_	CHA/DNA tetrahedron	CV, SWV, EIS	human serum	0.1–1000	0.026	Shuo et al. (2021)
VD_3_	Co-Ag/PANI-PPY/IL	CV, SWV, EIS	human serum and urine	12.5–22,500	7.3	Anusha et al. (2021)
PGE2	anti-PGE2/DSP	EIS	human urine	0.28–11.35		Ganguly et al. (2021)

1-NPP, 1-Naphthyl phosphate; DTSP, dithiobis (succinimidyl propionate); GA, glutaraldehyde; CNC, carbon nanotube; CNT, cellulose nanocrystal; CMK-3, ordered mesoporous carbon CMK-3; Cys, Cysteamine; DSP, thiol cross-linker.

### 3.1 Electrochemical sensors for the detection of steroid hormone

Current electrochemical sensing technology to detect steroid hormone is mainly used for early diagnosis of disease, pregnancy research, food toxicity and pollution levels (Kelch et al., 2020). The following is a review of the electrochemical sensors of progesterone, 17β-estradiol, testosterone, cortisol, and VD.

#### 3.1.1 Electrochemical sensors for the detection of progesterone

Progesterone is most commonly used as a pregnancy biomarker (Goh et al., 2016), which also has a central role in biology and medicine (Kanninen et al., 2019; Polat et al., 2020). Monitoring progesterone concentrations has been reported to be important in the autoimmune disease in women during menstruation (Chang and Wang, 2020), the dairy industry that can supply trustworthy data on mammalian reproduction by keeping track of progesterone levels (Yu and Maeda, 2017) and the environmental health protection by monitoring the progesterone in the wastewater (Cui et al., 2021).

Naderi and Jalali modified glassy carbon electrode (GCE) for progesterone accurately trace analysis in human serum samples and medicines using multi-wall carbon nanotubes (MWCNTs), AuNPs and poly-L-serine (Naderi and Jalali, 2020). DishaKumari et al. (2021) used magnetic graphene oxide (MGO) nanocomposite as an electrode material for the loading of bio-receptors. The increased surface area with strong electric conductivity improved sensor responsiveness. This design amplified the electrochemical signal and avoided the requirement of labeling enzymes and substrates.

Recently, progesterone detection has been accomplished using a variety of aptamer-based sensor designs. A photo-electrochemical sensor was created by Li et al. (2020) using magnetic-optical Fe_3_O_4_@SiO_2_@TiO_2_-NH_2_-aptamer-nanoparticles. The sensor could selectively capture progesterone in complex biological samples. Samie and Arvand prepared a label-free electrochemical progesterone aptamer sensor using graphene quantum dots-NiO-Au hybrid nanofibers/NH_2_ functionalized MWCNTs (GQDs-NiO-AuNFs/f-MWCNTs) and was successfully used to determine progesterone in human serum samples and pharmaceutical preparations (Samie and Arvand, 2020). Moreover, Zhu’s team simultaneously used antibodies and aptamer together to propose a progesterone sandwich assay, designing a sandwich-structured cathode photochemical biosensor (Zhu et al., 2020). The advantages of the sensor were easier progesterone determination, higher sensitivity and selectivity.

#### 3.1.2 Electrochemical sensors for the detection of estradiol

So far, multiple electrochemical sensors of estradiol were aiming for 17β-estradiol, which is a natural estrogen secreted by humans and domestic animals with the strongest estrogen activity. Even trace amounts of exogenous 17β-estradiol entering the body can cause significant damage to our health (Pu et al., 2019). So, effective 17β-estradiol monitoring is important.

In the research field of 17β-estradiol electrochemical sensor, composite nanomaterials made of carbon nanomaterials and other nanomaterials have been widely used. Tanrikut’s team prepared a highly efficient sensor to detect 17β-estradiol by using the NiFe_2_O_4_-mesoporous carbon (NiFe_2_O_4_-MC) nanocomposite which displayed an optimal electron transfer rate (Tanrikut et al., 2020). Supchocksoonthorn et al. designed a 17β-estradiol sensor employing carbon dots/polyaniline (CDs/PANI) composite (Supchocksoonthorn et al., 2021). 17β-estradiol and CDs/PANI are connected by hydrogen bonds and stacking to govern adsorption. Chang et al. demonstrated a split aptamer-based electrochemical estradiol aptamer sensor, with the first piece functionalized with adamantane and the second piece that had gold nanoparticles label (Chang et al., 2021). The disposable laser-scribed graphene electrode strip with exceptional sensitivity was successfully created from the sensing platform. In order to find 17β-estradiol in the eel Lee et al. (2020) serum, developed a screen-printed carbon electrode (SPCE) with 17β-estradiol-imprinted poly (aniline-co-metanilic acid) (EIPs) and tungsten disulfide (WS_2_). Da Silva and Pereira modified the electrode with MIP and carbon black (CB) to improve the sensitivity of the electrode by more than 173% compared with GCE (da Silva and Pereira, 2022). This electrochemical sensor could be easily fabricated and detect 17β-estradiol rapidly with a limit of detection (LOD) of 30 nM.

In addition to MIP electrochemical sensors based on carbon nanomaterials, Regasa and Nyokong created an electrochemical sensor based on MIP supported by AgNPs capped with 2-mercaptobenzoxazole (Regasa and Nyokong, 2022). The sensor was used to measure 17β-estradiol in actual water samples without the need of sample preconcentration processes, resulting in satisfactory selectivity, sensitivity, reusability and storage stability performances.

#### 3.1.3 Electrochemical sensors for the detection of testosterone

Testosterone is the most essential steroid released by testicular stromal cells (Gugoasa and Stefan-van Staden, 2018) whose levels are related to many male hormone disorders and cardiovascular diseases.

To create a testosterone-imprinted electronically conductive polymer (TIECP) on the sensing electrodes, Liu et al. optimized the synthetic self-assembly of poly (aniline-co-metanilic acid) and testosterone using an electrochemical method (Liu et al., 2020). This technique optimized the conductivity of nanomaterials. Moreover, Bulut’s team synthesized a new phenylenediamine-benzodithiophene polymer (pBDBT) used to manufacture biosensors for testosterone detection (Bulut et al., 2020). A platform for real-time field detection was provided by glutaraldehyde-fixed testosterone antibodies on the polymer-coated SPCE surface. It can be used for testosterone analysis in illicit drugs.

#### 3.1.4 Electrochemical sensor for the detection of cortisol

Cortisol is an important glucocorticoid found in a variety of biological fluids. Abnormally elevated cortisol levels can cause hypertension, damage to muscle tissue and immune system.

Now, more and more studies of electrochemical sensors for cortisol are being developed. For example, Vargas et al. (2020) developed a dual-electrochemical immunosensor based on gold microchip for the simultaneous detection of insulin and cortisol, which relied on competitive immunoassays with alkaline phosphatase (ALP) labeling . In addition, Liu et al. (2021) designed the electrochemical immunosensor for flexible AuNPs/MWCNTs/polydimethylsiloxane thin films and Naik et al. (2021) designed the “smart bandage” microfluidic platform sensor for graphene/silver solution. These three sensors are all used to detect cortisol and have the potential for instant applications.

In the study of cortisol molecularly imprinted polymers sensors, Tang et al. (2021). used a high permeability sweat-absorbing porous hydrogel to prepare a non-invasive, touch-based MIP electrochemical sensor Besides, poly glycidylmethacrylate-co ethylene glycol dimethacrylate (GMA-co-EGDMA) were used for the research of flexible MIP biosensor (Mugo and Alberkant, 2020). These two sensors have the advantages of being stretchable, small-portable and without sampling, and can serve as human wearable devices and instant application devices.

In addition to using a single biometric element to develop sensors, Huang et al. (2021) presented a highly sensitive and selective electrochemical sensor with an aptamer-antibody sandwich mode A specific combination of antibodies and aptamers was utilized to identify the target cortisol. Whereafter, Yu et al. (2022) suggested a novel electrochemical aptamer sensor using functionalized graphene (FG) and nitrogen-deqcarbon quantum dots (N-CQDs) integrating MIP techniques for trace analysis of cortisol in saliva samples.

#### 3.1.5 Electrochemical sensors for the detection of VD

VD is not only a lipid-soluble vitamin but also an immunomodulatory hormone that has two present forms of VD_2_ and VD_3_ (Charoenngam and Holick, 2020). As the best marker for the monitoring of VD levels, 25-hydroxy VD (25(OH)D) is frequently employed in clinical diagnosis (Binkley et al., 2010; Farrell and Herrmann, 2013).

In the field of VD electrochemical sensors based on carbon nanomaterials, Thangphatthanarungruang prepared a graphene nanocomposite sensor for simultaneously measuring vitamins (A, D, E and K) that are lipid-soluble in various matrix samples (infant milk, yogurt and parsley) (Thangphatthanarungruang et al., 2020). Anusha designed a label-free impedance sensor using ethyl-3-(3-dimethyl aminopropyl) carbodiimide-N-hydroxysuccinimide/graphitic carbon nitride-β-cyclodextrin (EDC-NHS/GCN-β-CD)@AuNPs composite to assay serum samples for the presence of 25(OH)D_3_ (Anusha et al., 2022). It is less destructive to its biomolecular activity, thus improving the sensitivity of detection. Kaur et al. (2021) designed a voltammetric immunosensor based on molybdenum sulfur MoS_2_/AuNPs/tin fluoride oxide with 25(OH)D_3_ as the target molecule The result showed that the MoS_2_-modified AuNPs model demonstrated excellent detection conductivity, sensitivity and stability.

Some electrochemical sensors using VD_3_ aptamer were also reported. Wadhwa’s team prepared a portable electrochemical aptamer sensor to identify VD_3_ employing graphene quantum dot-gold hybrid nanoparticles (GQD-Au) with a LOD of 0.70 nM (Wadhwa et al., 2020). Subsequently, Shuo et al. (2021) proposed a novel electrochemical aptamer sensor for sensitively detecting 25(OH)D_3_ by fixing DNA tetrahedra on Au surfaces and a technique for catalytic hairpin assembly (CHA) amplification.

In addition, Anusha’s group synthesized a new composite material based on polyaniline-polypyrrole (PANI-PPY) copolymer doped with silver-cobalt (Co-Ag) and ionic liquid (IL) (Anusha et al., 2021). The composite material was first used to modify the GCE. Later, a manual paper sensor was created using the suggested material. And using two different tests to detect VD_3_ in serum and urine samples.

### 3.2 Electrochemical sensors for the detection of fatty acid derivative hormone

The fatty acid derivative hormone is a metabolite produced by lipids through oxidative and enzymatic metabolic pathways including iso-prostaglandin and prostaglandin. Prostaglandin regulates pathological processes in female reproductive function (Niringiyumukiza et al., 2018), inflammation, and tissue repair (Rael, 2016). The lipid peroxidation biomarker 8-iso-prostaglandin F2α and carotid subclinical atherosclerosis showed a significant positive connection (Alharby et al., 2019).

In previous reports, electrochemical sensors of prostaglandin E1 (Zheng et al., 2016) and 8-iso-prostaglandin F2α (Sanchez-Tirado et al., 2017) have been successively reported. With the deepening understanding of the fatty acid derivative hormone and the development of electrochemical sensors, Ganguly et al. (2021) developed a three-electrode planar gold microelectrode system with flow-based nanopore membranes for electrochemical immunosensors for detecting prostaglandin E2 (PGE2). This sensor can be used in both clinical and home settings for a more immediate, fast and accurate diagnosis of urinary tract infection.

## 4 Conclusion and future perspectives

This paper mainly describes the recent research progress of electrochemical biosensors in the field of lipid hormone detection in the past 3 years. This could help the future development of such sensors in medicine and science. Furthermore, combined with the important role and significance of lipid hormones such as progesterone, estradiol, testosterone, cortisol, VD, prostaglandin in regulating human life activities and disease control, and the easy application of electrochemical sensors, the development of lipid hormone electrochemical sensors has attracted more and more attention in recent years.

Overall, so far, there are more comprehensive and mature studies of steroid electrochemical sensors than those of fatty acid derivative. Current electrochemical biosensors for fatty acid derivative hormone use only antibodies as recognition elements. In the future, electrochemical sensors will have considerable potential for development and applications in the detection of lipid hormone. A key opportunity for the development of electrochemical sensor and biosensor platforms is the introduction of innovative functional nanomaterials and analytical technologies. Such as new electrode materials with more selectivity and sensitivity, more portable wearable sensors and instant application sensors still need to be continuously explored by researchers.
